# Small Molecules: Therapeutic Application in Neuropsychiatric and Neurodegenerative Disorders

**DOI:** 10.3390/molecules23020411

**Published:** 2018-02-13

**Authors:** Stefania Schiavone, Luigia Trabace

**Affiliations:** Department of Clinical and Experimental Medicine, University of Foggia, Via Napoli, 20, 71122 Foggia, Italy; luigia.trabace@unifg.it

**Keywords:** small molecules, neuropsychiatric disorders, neurodegenerative disorders, redox modulation, oxidative stress

## Abstract

In recent years, an increasing number of studies have been published, focusing on the potential therapeutic use of small catalytic agents with strong biological properties. So far, most of these works have only regarded specific clinical fields, such as oncology, infectivology and general pathology, in particular with respect to the treatment of significant inflammatory processes. However, interesting data on possible therapeutic applications of small molecules for the treatment of neuropsychiatric and neurodegenerative illnesses are emerging, especially with respect to the possibility to modulate the cellular redox state. Indeed, a crucial role of redox dysregulation in the pathogenesis of these disorders has been widely demonstrated by both pre-clinical and clinical studies, being the reduction of the total amount of free radicals a promising novel therapeutic approach for these diseases. In this review, we focused our interest on studies published during the last ten years reporting therapeutic potential of small molecules for the treatment of neuropsychiatric and neurodegenerative disorders, also based on the biological efficiency of these compounds in detecting intracellular disturbances induced by increased production of reactive oxygen species.

## 1. Aims and Methodology for Literature Search

Small molecules are crucial players in several chemical reactions, able to detect the presence of a copious number of cellular metabolites [1]. Indeed, in a large variety of pathological conditions, several molecular alterations, which do not generally occur in physiological states, are detectable and mainly regard the increased production of free radicals, pH alterations and impaired generation of some specific biomolecules [2]. In recent years, an increasing number of studies focused on the potential therapeutic use of small agents with strong biological properties have been published [3], but, so far, most of the reports on this subject have mainly regarded proliferative or infectious diseases [4,5,6,7,8,9,10,11,12,13,14,15,16].

Here, we aimed to review studies published during the last ten years (from 1 November 2007 to 1 November 2017), focused on the potential therapeutic use of small agents for the treatment of neuropsychiatric and neurodegenerative disorders, in particular with respect to their bioactivity of sensoring intracellular alterations caused by enhanced free radical amount, discussing the benefits and the limits of this therapeutic approach.

The literature source for this review included open access papers found in Pubmed in the above-mentioned period using, as key words, the term small molecules in combination with the following words: neuropsychiatric disorders, neurodegenerative diseases, schizophrenia, mood disorders, anxiety, autism, Alzheimer’s disease, Parkinson’s disease, amyotrophic lateral sclerosis, stroke, spinal cord injury, traumatic brain injury. Each of these combinations has been further associated with the terms: oxidative stress, redox modulation, ROS, free radicals. By using these keyword combinations, we obtained a total number of 158 papers, with 14 duplicate records, which were removed for further screening. We then excluded 42 papers which were not written in English or were other than original research papers or reviews. A second round of exclusion concerned papers (29) which did not provide a detailed description of the chemical characteristics of the considered small molecules. Finally, 73 papers were included in the analysis. The total number of references of this paper also included the ones used for the introducing statements of the different sections of the manuscript. 

## 2. The Use of Small Molecules in Neuropsychiatric Disorders

**Figure 1:**
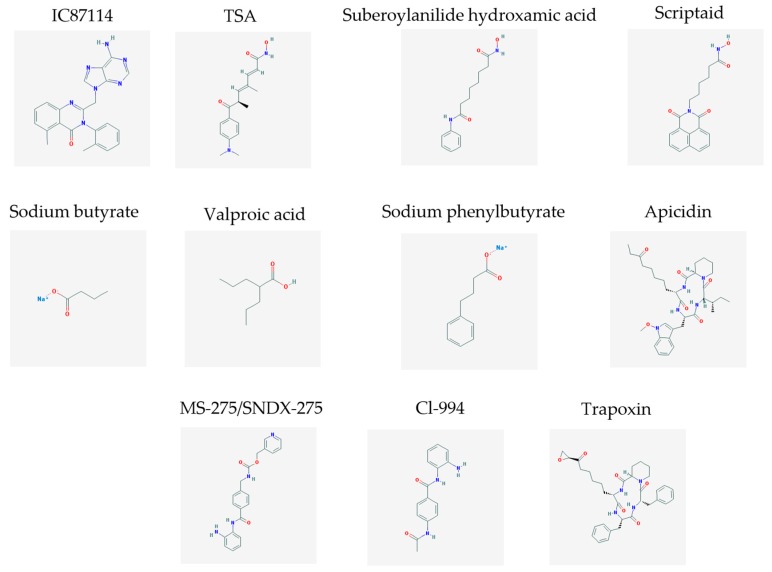
Chemical structure of small molecules with therapeutic potential in neuropsychiatric disorders.

The main findings related to the possible therapeutic potential of small molecules in the treatment of schizophrenia, mood disorders, anxiety and autism are reported in Table 1. The chemical structures (https://pubchem.ncbi.nlm.nih.gov/) [17] of specific small molecules with therapeutic potential in neuropsychiatric disorders are shown in Figure 1.

## 3. The Use of Small Molecules in Neurodegenerative Disorders

### 3.1. Therapeutic Potential for Alzheimer’s Disease

A crucial event in the pathogenesis of Alzheimer’s disease (AD) is represented by the amyloid-β peptide (Aβ) aggregation which initiates a cascade of molecular pathways, finally resulting in neuronal death and degeneration [28]. Importantly, abnormal Aβ metabolism can be detected several years before AD onset [29] and this aspect represents an important pharmacological target for early therapeutic interventions. However, so far, no compound specifically targeting the process of Aβ accumulation, and mainly developed by using animal models of the disease, has been translated into clinical practice [30]. Therefore, the identification of small molecules, acting on Aβ accumulation and aggregation, has represented the focus of an increasing number of studies in this field, in the perspective of opening a novel and promising “chapter” in the history of compounds to be used in AD. One of the most complete and straightforward works in this sense is represented by a very recent paper by Habchi and co-workers, in which the authors reported the systematic development of some small molecules, classified as “set A” (seven molecules showing a similar or greater effect than the bexarotene) and “set B” (five molecules able to totally inhibit Aβ42 aggregation for a period of at least 10 h), that inhibit specific steps of Aβ42 aggregation [31]. However, for the sake of clarity, it should be specified that some of the small molecules identified in this work, such as MM11253 and adapalene, were previously described by other authors to significantly impact not only the onset of the aggregation but also Aβ42 oligomer proliferation [32,33].

The other records found with our research strategy, highlight, rather, the development of small molecules targeting another crucial pathogenetic process in AD progression, i.e., the loss of metal ion homeostasis and their impaired compartmentalization [34,35], especially concerning the redox active Fe(II/III) and Cu(I/II), that have been found as highly concentrated in senile plaques [36,37], in the cortex and hippocampus [38,39]. Hence, these reactive metals can bind to Aβ species, then undergoing Fenton reaction, resulting in the production of specific reactive oxygen species (ROS), such as hydrogen peroxide and hydroxyl radical, which may facilitate Aβ aggregation and trigger neurodegeneration [35]. Therefore, a valuable therapeutic strategy to reduce neurotoxicity, induced by the interaction between metals and Aβ species, and to re-establish metal ion homeostasis in the brain, might be represented by the metal-Aβ association outbreak via metal chelation. So far, the most used chelators in AD therapy included ethylenediaminetetraacetic acid (EDTA), clioquinol and PBT2, an 8-hydroxyquinoline derivative. In the attempt to translate preclinical findings to possible clinical application, the last two compounds were also tested in phase II clinical trials and they have been reported to significantly improve cognitive functions [40,41], despite their serious side effects, such as the subacute myelo-optic neuropathy induced by clioquinol. Consistent efforts have been also addressed to the development of small molecules, such ascyclen, KLVFF peptide, curcumin, IMPY, and p-I-stilbene that were able to synergistically link both metal ions and Aβ [42,43,44], in order to overcome the limit of the previously developed metal chelating compounds, especially related to blood-brain barrier permeability.

### 3.2. Therapeutic Potential for Parkinson’s Disease

Parkinson’s disease (PD) is a neurodegenerative disorder characterized by the loss of the dopaminergic neurons in the substantia nigra. Several pathogenetic mechanisms have been proposed for this Central nervous system (CNS) disorder. One of the most investigated is related to mitochondrial and redox dysfunctions [45,46], including mutations in the mitochondria-specific kinase PTEN-induced kinase 1 (PINK1), as well as in the E3 ubiquitin ligase Parkin, a mitochondria-associated protein [47]. In line with this concept, several papers reported the therapeutic potential of PINK1/Parkin pathway activation in PD [48,49]. However, despite the widely known mechanism of small molecule-mediated activation of kinases, typically accomplished by binding their allosteric regulatory sites, PINK1 has been shown to not contain small molecule-binding sites, being, therefore, its pharmacological activation mediated by other mechanisms [49]. On the other hand, mildronate [3-(2,2,2-trimethylhydrazinium) propionate dihydrate], a small molecule with charged nitrogen and oxygen atoms that protect mitochondria, has been described to act as neuroprotective compound in a mouse model of neurotoxicity induced by azidothymidine, via suppression of brain inflammation and apoptosis, as well as decrease of cytochrome oxidase c, caspase-3, inducible Nitric Oxide Synthase (iNOS) and cellular apoptosis susceptibility-protein [50]. The mildronate-related neuroprotection was further confirmed in a study performed by using a rat model of PD that was obtained by unilateral intra-striatal injection of the neurotoxin 6-hydroxydopamine (6-OHDA). Indeed, mildronate administration to 6-OHDA-injected animals prevented the loss of specific biomarkers, such as tyrosine hydroxylase, ubiquitin and Notch-3, which are known to assure neural and glial integrity, simultaneously decreasing the expression of specific markers of inflammation, such as iNOS [51]. In line with mitochondria dysfunctions, another crucial pathogenetic mechanism associated to the development of PD is related, at molecular levels, to dysfunctions of GTPases, especially the ones regulating the dynamic processes of mitochondria fission and fusion, the organelle transport along axons, the axon maintenance, as well as the neuronal survival, and to neuroinflammation and oxidative stress enhancement [52]. With respect to redox imbalance, the small GTPase Rac1, which crucially regulates the functioning of the free radical producer NOX1, has been reported to accumulate in dopaminergic neurons of patients affected by PD [53]. Interestingly, a library containing 5 million small molecules has been probed as modulator for different GTPases, such as Rab5, Rab7, Cdc42, wild type Ras and mutant Ras, Rho, and Rac that selectively activate GTPase subfamilies [52].

Another significant acquired knowledge in the field of molecular mechanisms leading to PD, deals with the role of specific proteins, including α-synuclein. Indeed, in a very interesting paper of Misook and co-workers, authors attempted to synthesize a library of small molecules, in order to rapidly and efficiently identify potential pharmacological “chaperones”, i.e., small molecules that bind proteins and stabilize them against degradation, as well as novel chaperone inhibitors against α-synuclein [54].

**Figure 2:**
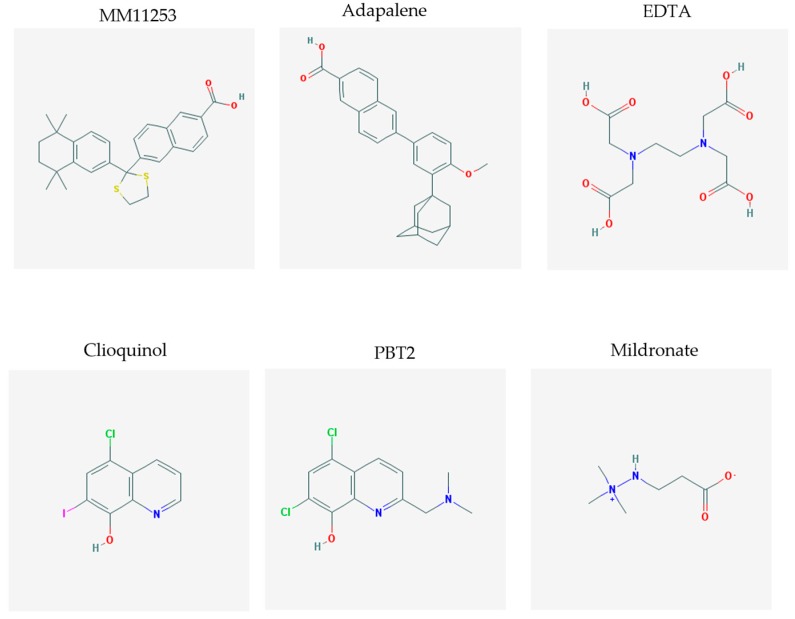
Chemical structure of small molecules with therapeutic potential in Alzheimer’s or Parkinson’s diseases.

The chemical structures (https://pubchem.ncbi.nlm.nih.gov/) [17] of small molecules with therapeutic potential in AD and PD are shown in Figure 2.

### 3.3. Therapeutic Potential for Amyotrophic Lateral Sclerosis

Amyotrophic lateral sclerosis (ALS) is a fatal neurodegenerative disease, characterized by motor neuron death and rapid progression, for which there are no effective therapeutic opportunities. From a pathogenetic point of view, 20% of the familiar forms of ALS are caused by mutations in the SOD1 gene [55] which induce a gain of toxic functions by motor neurons. The exact cause of ALS is still unknown. However, cell autonomous and non-cell autonomous mechanisms are reported to contribute to the degenerative process [56,57,58]. More recent research has identified TAR-DNA binding protein-43 (TDP-43) as a crucial player in the pathogenesis of both sporadic and non-SOD1 familial ALS [59,60]. Furthermore, several pathogenic initiators of ALS, including oxidative stress, mitochondrial dysfunctions, neuroinflammation and depletion of neurotrophins have been identified [61]. Despite the limited therapeutic efficacy of the current available treatments, in recent years a significant number of studies described the development and/or proposed the use of different small molecules for ALS (Table 2).

## 4. The Use of Small Molecules in Neurological Disorders Associated with Neurodegeneration

### 4.1. Therapeutic Potential for Stroke

**Figure 3:**
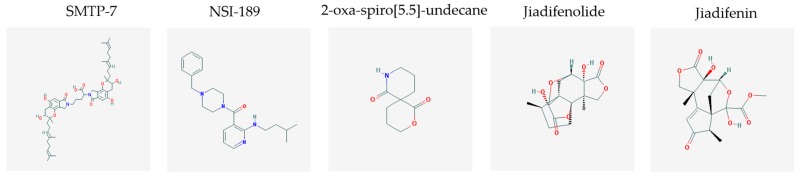
Chemical structure of small molecules with therapeutic potential in neurodegeneration associate to stroke.

Stroke is a neurological disorder commonly associated with a very high mortality and disability rate, for which the only existing therapeutic opportunity consists in the use of a thrombolytic agent in order to restore the blood flow in the ischemic area [76]. However, only a limited number of subjects can benefit from this pharmacological intervention. Therefore, there is an urgent need to develop new therapeutic strategies. In this context, the potential use of small molecules, as possible pharmacological choice, has become a field of lively scientific research. In particular, the therapeutic effects of the small molecule *Stachybotrys microspora* triprenyl phenol-7 (SMTP-7), that promotes activation of plasminogen through the modulation of plasminogen conformation [77], against cerebral ischemia has been tested in several rodent models of stroke [78,79,80]. Importantly, together with its strong thrombolytic properties, SMTP-7 has been also described as a decrease of neuroinflammation related to stroke-induced neurodegeneration [78,81]. Despite the utility of data obtained on rodent models of stroke regarding this small molecule, studies in higher-order species, such as primates, whose cerebral vascularization and vessel morphology show more significant similarities with the human counterpart than rodents [82], can be considered extremely significant for a more direct translation towards the human pathology and for a potential clinical application of SMTP-7. In this perspective, the study conducted by Sawada and co-workers appears of great importance. Indeed, authors succeeded in demonstrating in primates, by using the photochemically induced thrombotic middle cerebral artery occlusion model, causing cyclic flow reductions typical of infarct progression in stroke patients, that SMTP-7 reduced cerebral infarction, neurologic deficits, and hemorrhage in the infarct area, thus also displaying a significant neuroprotection [83]. Neuroprotective effects of small molecules against stroke-associated neurodegeneration, especially in terms of neurostructural benefits and enhancement of neurogenesis, have been also clearly reported in a recent elegant work that evaluated the effects of an oral administration of the small molecule NSI-189, which is already in clinical trial for the treatment of major depression and prevention against suicide (https://clinicaltrials.gov/) [84], in the middle cerebral artery occlusion mouse model [85]. Thanks to a well-conceived experimental approach, authors were able to demonstrate that NSI-189 promoted behavioral recovery, enhanced cell proliferation and neurogenesis, and upregulated specific neurogenic factors, such as BDNF, if administered at a wider therapeutic window of 6 hours after stroke [85]. Although no data on the effects of this small molecule on primate models of stroke are currently available, results obtained on the mouse model, could be certainly considered very promising in the perspective of approving this small agent also for the treatment of stroke in humans. Another promising category of pharmacological compounds which recently raised interest in the research of novel therapeutic options for stroke and associated neurodegeneration is represented by natural product-based small molecules with neurotrophic, neurogenic and anti-neuroinflammatory actions. Recent efforts in this direction by the group of Jhelum and collaborators led to the discovery of some novel compounds based on 2-oxa-spiro[5.5]-undecane, derived from the natural product paecilomycine A, which have been described as booster of neurite growth and neuronal regeneration, and as a neuroinflammation decreaser [86,87]. Other natural small molecules, such as jiadifenolide, jiadifenin and (1*R*,10*S*)-2-oxo-3,4-dehydro-xyneomajucin (ODNM), have been also tested in vitro for their neuritogenic and neurotrophic activity, by using primary rat hippocampal neurons and ESC-derived motor neurons [88] or PC12 cells [89]. The chemical structures (https://pubchem.ncbi.nlm.nih.gov/) [17] of small molecules with therapeutic potential for stroke-related neurodegeneration are shown in Figure 3.

### 4.2. Therapeutic Potential for Spinal Cord Injury

Our research strategy allow us to identify 19 records focused on the potential therapeutic use of small molecules in spinal cord injury and on the possible beneficial effects on its associated neurodegeneration [90] (Table 3).

### 4.3. Therapeutic Potential for Traumatic Brain Injury

Traumatic brain injury is commonly considered an important cause of morbidity and mortality. This pathological event has been described as the initiator of a cascade of detrimental processes that can exacerbate the primary injury, worsen long-term outcome and increase the risk of neurodegenerative complications [110]. Several pathogenetic factors have been considered as crucial players in this mechanism. Among them, the most investigated one refers to mitochondria dysfunctions, with enhanced ROS generation and consequent oxidative stress and overproduction of pro-inflammatory cytokines, finally resulting in inflammation, edema, blood brain barrier loss of integrity and increased permeability, neurotoxicity, and cell death [111]. Thus, the development of small molecules that target each of these processes is the focus of an increasing interest, in order to find alternative therapeutic strategies against complications derived from neurodegeneration. With respect to mitochondria dysfunctions, a recent report of Wu and co-workers investigated the possible beneficial role of Mdivi-1, a small molecule inhibitor of the key mitochondrial fission protein dynamin-related protein 1(Drp1), against neuronal death and functional outcome deficits, in a mouse model of traumatic brain injury. By this interesting experimental approach, authors demonstrated that Drp1 inhibition, obtained by Mdivi-1 administration, significantly alleviated behavioral alterations, brain edema, impairment of mitochondrial morphology and cell death induced by traumatic brain injury [112].

Evaluation of the potential therapeutic effects of small molecules that reduce oxidative stress has been also conducted by Wang and Co-workers, who demonstrated the neuroprotective effects of edaravone, a synthetic free radical scavenger small molecule, in a rat model of traumatic brain injury. Indeed, administration of this compound 2 and 12 h after traumatic brain injury significantly decreased neuronal loss and death, and reduced oxidative stress, also acting on non-neuronal cell types, such as astrocytes and microglia. Furthermore, this compound was also shown to positively act on blood brain barrier loss of integrity and altered functioning, as well as to decrease the production of inflammatory cytokines [113].

In line with the evaluation of small molecules active on neuroinflammatory processes induced by traumatic brain injury, it has been reported that administration of MW01-2-151WH (MW151), a small molecule inhibiting the production of proinflammatory cytokines, such as interleukin-1 beta (IL-1β) and tumor necrosis factor alpha (TNFα) but not blocking the release of anti-inflammatory cytokines, such as interleukin-10 (IL-10), was able to suppress cytokine acute up-regulation and downstream cognitive impairment [114,115].

Another molecular player which has been described as a crucial component in the pathologic process leading to neuronal death and neurodegeneration following traumatic brain injury is Toll-like receptor 4 (TLR4). Therefore, the development of small molecules targeting this element might represent an innovative therapeutic strategy. In this context, administration of resatorvid, a small molecule considered as inhibitor of TLR4-mediated pathways, has been shown to dramatically attenuate neuronal apoptosis associated to traumatic brain injury, to significantly decrease TNF-α and IL-1 β levels and to improve neurological recovery [116]. As it has been verified that TLR4 expression was also significantly increased in human contusion specimens after traumatic brain injury [116], resatorvid and other small molecules inhibiting pathogenetic events mediated by TLR4 might represent promising candidates for therapy following traumatic brain injury in humans.

**Figure 4:**
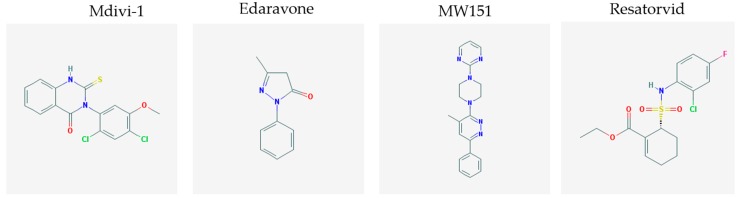
Chemical structure of small molecules with therapeutic potential in neurodegeneration associate to traumatic brain injury.

The chemical structures (https://pubchem.ncbi.nlm.nih.gov/) [17] of small molecules with therapeutic potential for traumatic brain injury are shown in Figure 4.

## 5. Discussion and Conclusions

In this review, we have provided an extensive picture taken from the scientific literature of the last ten years dealing with the “hot topic” of potential therapeutic use of small molecules in the treatment of neuropsychiatric and neurodegenerative disorders. A key element of our work is that, in our research strategy, we also included keywords referred to neurological disorders, such as stroke, spinal cord and traumatic brain injury, which are major concerns for current day increased death rate and closely associated with neuronal death and degeneration.

From a critical reading of the papers cited in this review, specific discussion points may arise. In our opinion, one of the most important ones is related to the fact that most of the identified small molecules have been mainly tested for their therapeutic potential in animal models of the disease and, most of the time, in rodents (mice or rats). Despite the scientific importance of animal models in the progress of the understanding of pathogenetic pathways leading to a specific brain disease and their crucial contribution to the identification of specific pharmacological targets, a “too direct” transfer of data obtained on animal models of neuropsychiatric and neurodegenerative disorders to the human pathology may result in a risky process, leading to the generation and dissemination of misleading and biased information. Moreover, especially with respect to data concerning specific small molecules that have been reported to improve cognitive symptoms in some rodent models, a critical aptitude should be kept, considering that all the deep and complex emotional aspects, which could have a significant impact on cognitive alterations related to neuropsychiatric and neurodegenerative diseases in humans, are quite totally lacking in rodent models. Another important aspect that enables a more direct translation of rodent data to humans is related to a significant lack of clear and detailed information about the real ability of small molecules showing a therapeutic potential on rodent models of neuropsychiatric and neurodegenerative diseases to cross the blood brain barrier and to be effectively delivered to the CNS. This is still more complicated by further limitations related to the real usefulness of the available in vitro models of blood brain barrier. Globally, all these concerns may contribute to explain the facts that some small molecules, which were finally selected for clinical trials and found to improve cognitive dysfunctions also in humans (such as clioquinol and PBT2), were burdened by very serious side-effects. In conclusion, there is an urgent need to enhance the effort in conceiving rigorous researches on the possible therapeutic use of small molecules in neuropsychiatric and neurodegenerative disorders, also considering the several still “unexplored” fields of these diseases.

## Figures and Tables

**Table 1:** The use of small molecules in neuropsychiatric disorders.

Neuropsychiatric Disorder	Small Molecules	Mechanism of Action	Effects	References
Schizophrenia	IC87114	Inhibition of phosphoinositide 3-kinase subunit, p110δ	- Block of the effects of amphetamine in a mouse pharmacological model of psychosis - Reversal of schizophrenia-related phenotypes in the rat neonatal ventral hippocampal lesion model	[18]
	Phosphodiesterase 10A inhibitors	Inhibition of Phosphodiesterase 10A	- Activation of the cAMP/PKA signalling in the basal ganglia - Potentiation of dopamine D_1_ receptor signalling - Inhibition of dopamine D_2_ receptor signalling - Improvement of positive, cognitive and negative symptoms of schizophrenia (animal models)	[19]
	Histone deacetylase 1 (HDAC1) inhibitors	Inhibition of HDAC1	- Improvement of cognitive disturbances, negative symptoms and low motivation	[20,21]
Mood disorders	HDAC inhibitors (TSA, suberoylanilide hydroxamic acid, scriptaid, derivatives of aliphatic acid such as sodium butyrate, sodium phenylbutyrate, and valproic acid. Cyclic tetrapeptides such as apicidin, trapoxin, depsipeptide (FK-228)/romidepsin and benzamides such as MS-275/SNDX-275 and Cl-994)	Inhibition of HDAC1 and 2	- Inhibition of HDAC - Reversal/block of increased histone deacetylation, DNA methylation, and hypothalamic-pituitary-adrenal stress responses in rodent models of depression associated with early life events - Induction of antidepressant-like effects - Protection of neurons against oxidative stress-induced neuronal death - Induction of Brain-derived neurotrophic factor (BDNF) and Glial-derived neurotrophic factor release (GDNF)	[22,23,24,25]
Anxiety	HDAC inhibitors	Inhibition of HDAC1 and 2	- Upregulation of BDNF exon I and IV mRNA expression in the prefrontal cortex - Enhancement of initial learning in contextual fear conditioning - Neuroprotection - Protection of cortical neurons against oxygen and glucose deprivation - Enhancement of GDNF and BDNF expression in astrocytes	[22,26]
Autism	Cdc2-like kinase 2 (CLK2) inhibitors	Inhibition of CLK2	- Decrease of synaptic deficits in neurons derived from patients with symptoms of autism spectrum disorder - Restore of normal sociability in an animal model of autism	[27]

**Table 2:** Small molecules developed and proposed for ALS treatment.

Small Molecule	Chemical Structure (https://pubchem.ncbi.nlm.nih.gov/) [17]	Mechanism of Action	Effects	References
1,10-Phenanthroline monohydrate	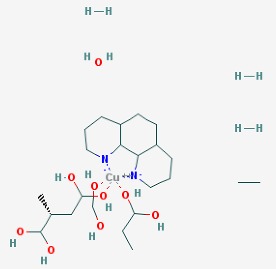	Matrix metalloproteinase inhibitor	- Increased motor neuron survival - Extension of ALS mice survival	[62,63]
CP55940	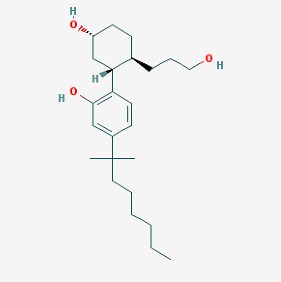	Cannabinoid receptor agonist	- Increased motor neuron survival	[63,64]
MDL 28170	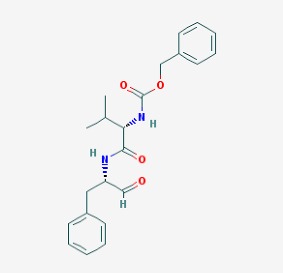	Calpain inhibitor	- Increased SOD1G93A/HB9::GFP motor neuron survival - Increased lifespan of SOD1G93A mice	[63,65]
77636 Hydrochloride and 3-tropanylindole-3-carboxylate methiodide	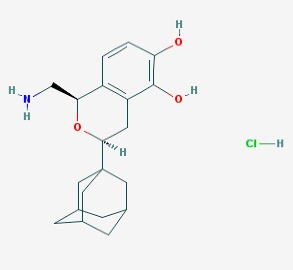 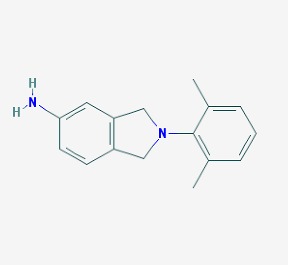	Ligands for neurotransmitter receptors	- Increased motor neuron survival	[63,66]
FPL-64176	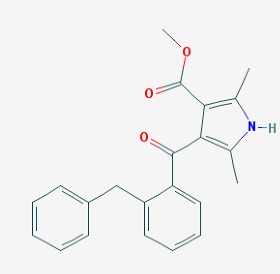	Calcium agonist	- Increased motor neuron survival	[63,66]
Tyrphostin A9	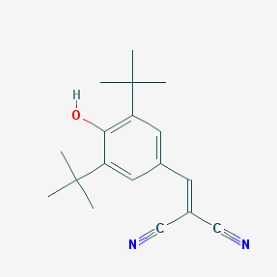	Multi-kinase inhibitor	- Increased survival of HB9::GFP motor neurons	[63]
Kenpaullone	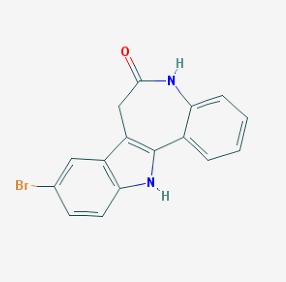	Inhibitor of GSK-3, CDK1/cyclin B, CDK2/cyclin A, CDK2/cyclin E and CDK5/p25	- Increased survival of motor neurons but not production of new motor neurons - Promotion of motor neuron survival when death is initiated by other types of stimuli - Promotion of long-term survival of wild type and mutant motor neurons in the presence or absence of trophic factors - Support of the neuronal structure of motor neurons - Preservation of morphological synapses of motor neurons - Maintenance of neuronal health when trophic factors are removed - Decrease of mutant SOD1 levels - Promotion of the survival of motor neurons derived from human ALS induced pluripotent stem cells	[63]
(±)-*trans*-1,2-bis(mercaptoacetamido)cyclohexane	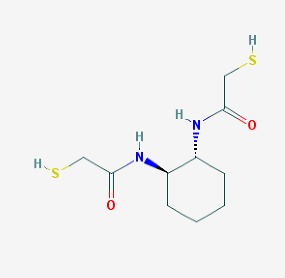	Mimic of the protein disulphide isomerase active site	- Protection against mutant SOD1 inclusion formation	[67]
CPN-9 (*N*-(4-(2-pyridyl)(1,3-thiazol-2-yl))-2-(2,4,6-trimethylphenoxy) acetamide)	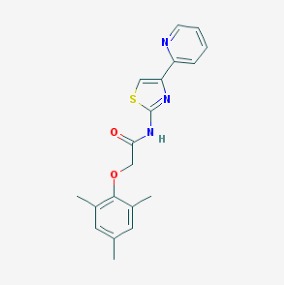	- Selective suppression of oxidative stress-induced cell death in a cell-type-independent manner - Upregulation of NF-E2-related factor 2 - Upregulation of heme oxygenase-1 - Upregulation of NAD(P)H quinone oxidoreductase 1 - Upregulation of glutamate–cysteine ligase modifier subunit	- ROS-dependent activation of the Nrf2 signalling pathway - Motor function maintenance - Delay of disease progression after onset	[68]
L-745,870	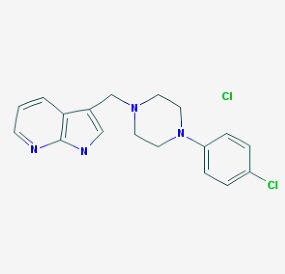	D4 receptor antagonist	- Inhibition of oxidative-stress-induced cell death - Upregulation of neuronal apoptosis inhibitory protein (NAIP/BIRC1) - Delay of symptom onset - Delay of weight loss and motor dysfunction - Reduction in the loss of neurons - Decreased activation of microglial cells	[69]
Bromocriptine	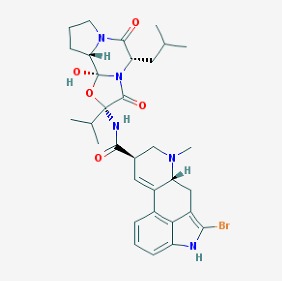	D2 receptor agonist	- Upregulation of antioxidant proteins (ATF-3 and HO-1) - Delay of disease progression - Improvement of motor functions - Increase of the post-onset survival of SOD1 H46R animals	[70]
CDDO-EA (2-cyano-3,12-dioxoolean-1,9-dien-28-oic acid-ethylamide)	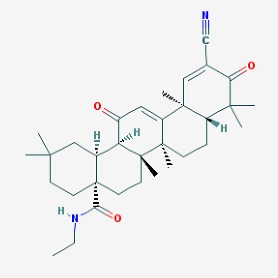	Activation of Nrf2/ARE signalling	- Increase in Nrf2 expression and nuclear localization - Increase in the levels of Nrf2-regulated antioxidant genes in mouse spinal cords - Reduction of weight loss and motor decline, and increase in lifespan	[71]
CDDO-TFEA (CDDO-trifluoroethylamide)	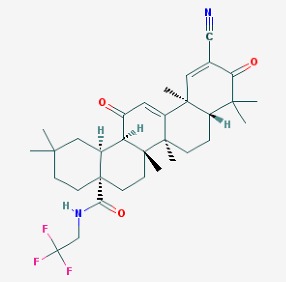	Activation of Nrf2/ARE signalling	- Increased expression of Nrf2 and the Nrf2 regulated genes, NAD(P)H quinine oxidoreductase, heme oxygenase-1 and glutathione reductase - Increased nuclear translocation of Nrf2 in primary rat neurons - Increase in Nrf2 expression and nuclear localization - Increase in Nrf2-regulated antioxidant genes in mouse spinal cords - Reduction of weight loss and motor decline, and increase in lifespan	[71]
RG108	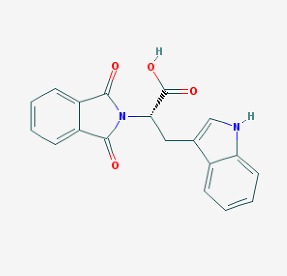	Noncovalent block of Dnmt active site and consequent Dnmt inhibition	- Prevention of DNA methylation accumulation in motor neurons and of their degeneration	[72]
Pyrazolone	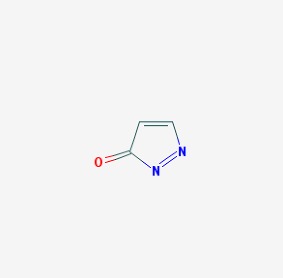	- Activation of the 26 proteosome subunit 4 and 6B - Activation of T-complex protein 1	- Neuroprotection	[73]
Trichostatin A	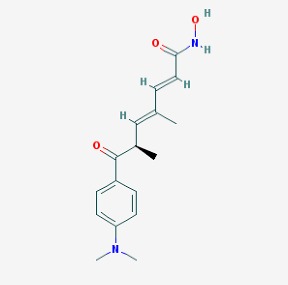	Histone deacetylase inhibitor	- Increased performance on rotarod tests - Improved stride length - Extended lifespan - Attenuation of astrogliosis and neuron loss in the lumbar spinal cord	[74]
Dichloroacetate	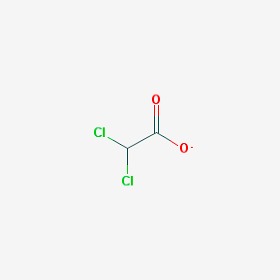	- Inhibition of the pyruvate dehydrogenase enzyme - Modulation of mitochondrial activity	- Reduction of astrocyte reactivity and motor neuron death - Prolonged lifespan by two weeks	[75]

**Table 3:** Small molecules developed and proposed for spinal cord injury.

Small Molecule	Chemical Structure (https://pubchem.ncbi.nlm.nih.gov/) [17]	Mechanism of Action	Effects	References
Ibuprofen/indomethacin	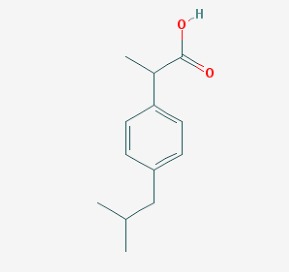 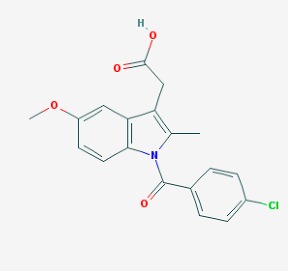	Inhibition of Rho-mediated pathways	- Improvement of motor recovery - Reduction of neuropathic pain - Increase of axonal sprouting and of the density of nerve fibers - Neuroprotection	[91,92,93,94,95,96,97,98,99]
Potassium bisperoxo (1,10-phenanthroline)oxovanadate	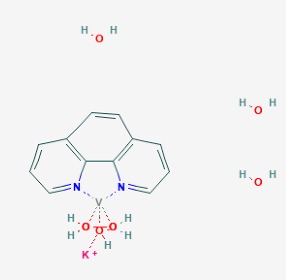	Protein tyrosine phosphatase inhibitor	- Improvement and normalization of sensorimotor functions in an animal model - Axonal protection - Rescue of sensory-evoked potentials - Rescue of blood vessels - Reduced apoptosis of cultured endothelial cells	[100]
RGFP966	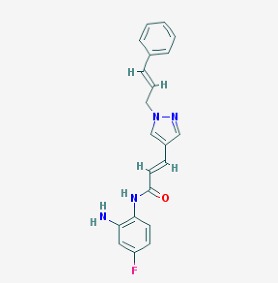	Block of histone deacetylase 3	- Neuroprotection via suppression of inflammation - Improvement of functional recovery	[101]
BIO5192	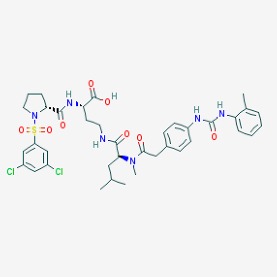	Inhibition of α4β1 integrin	- Decreased expression of the oxidative enzymes gp91phox, iNOS and cyclooxygenase-2 - Decrease of lipid peroxidation - Improvement of motor function - Decreased mechanical allodynia	[102]
Necrostatin-1	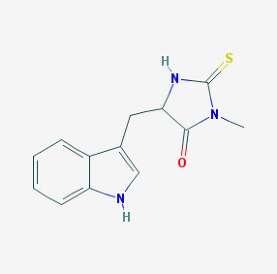	Inhibition of necroptosis targeting receptor-interacting protein kinase 1	- Reduction of ultrastructural damage to the endoplasmic reticulum and mitochondria - Inhibition of the expression of ERS-related genes and proteins after lesioning	[103]
Trimebutin	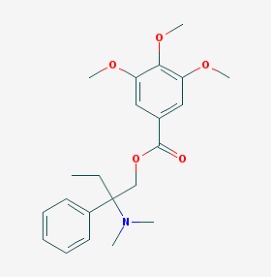	Agonism of adhesion molecule L1	- Improvement of ground locomotion - Enhancement of hindlimb locomotor functions - Reduction of areas and intensity of glial fibrillary acidic protein immunoreactivity - Increased regrowth of axons	[104]
Tacrine	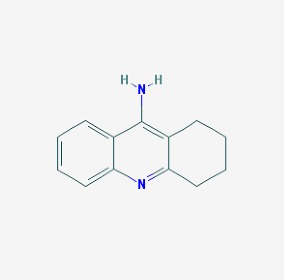	Agonism of adhesion molecule L1	- Rapid recovery of locomotor activities - Enhancement of regrowth of axons and myelination - Reduced astrogliosis	[105]
7,8-Dihydroxyflavone	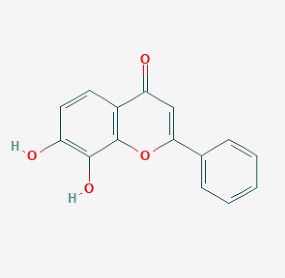	TrkB Agonism	- Protection of immature neurons from excitotoxicity-mediated death in vitro - Reduction of the death of adult-born immature neurons in the hippocampus - Prevention of Dendrite Degeneration	[106,107]
Tegaserod	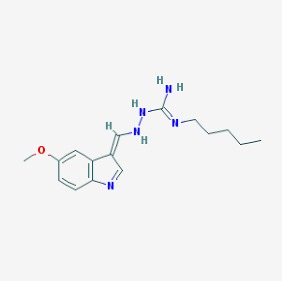	Mimetism of polysialic acid	- Promotion of hindlimb motor function - Increased numbers of neurons - Decreased glial fibrillary acidic protein immunoreactivity - Increased axonal density	[108]
LM11A-31	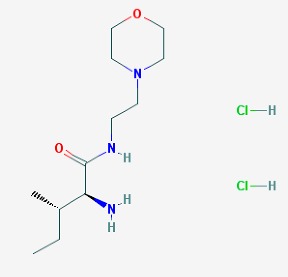	Block of proNGF Binding to p75	- Promotion of functional recovery - Improvement of motor function and coordination	[109]
